# First Complete Genome Sequence of *Escherichia albertii* Strain KF1, a New Potential Human Enteric Pathogen

**DOI:** 10.1128/genomeA.00004-14

**Published:** 2014-01-30

**Authors:** Krzysztof Fiedoruk, Tamara Daniluk, Izabela Swiecicka, Emilia Murawska, Malgorzata Sciepuk, Katarzyna Leszczynska

**Affiliations:** a Department of Microbiology, Medical University of Bialystok, Bialystok, Poland; b Department of Microbiology, Institute of Biology, University of Bialystok, Bialystok, Poland

## Abstract

*Escherichia albertii* has been recently recognized as an emerging human and bird enteric pathogen. Here, we report the first complete chromosome nucleotide sequence of a clinical isolate of *E. albertii* strain KF1, which may provide information about the pathogenic potential of this new species and the mechanisms of evolution of enteropathogenic *Escherichia* spp.

## GENOME ANNOUNCEMENT

*Escherichia albertii* is a new emerging enteric pathogen that has been associated with infections in humans and birds (1). This bacterium was first isolated from five Bangladeshi children with diarrhea, but it was initially identified as *Hafnia alvei* (2). However, further analysis has showed that those isolates were more closely related to the genera *Escherichia* and *Shigella*, and subsequently the isolates were reclassified in 2003 as *E. albertii* (3, 4). *E. albertii* is sometimes misidentified because it shares some virulence factors (e.g., intimin) with enteropathogenic *Escherichia coli* (EPEC) and enterohemorrhagic *E. coli* (EHEC) strains (5). Nevertheless, its pathogenic potential is poorly understood, and little is known about its prevalence in food and clinical samples. To date, only three draft genome sequences of *E. albertii* are available in GenBank, including the annotated scaffold representing the chromosome of *E. albertii* TW07627, originally isolated from children in Bangladesh.

Here, we report the complete chromosome sequence of *E. albertii* strain KF1, isolated together with *Campylobacter jejuni* and *C. difficile* from a stool sample from a 1-year-old child with acute diarrhea and identified according to Hyma et al. (6). The stool sample was collected with consent according to Polish law and approved by the Bioethics Commission of the Medical University of Białystok.

The genome of *E. albertii* strain KF1 was sequenced using three platforms, (i) an Ion Torrent 316 yielding 3,472,976 reads with an average length of 183 bp, (ii) an Illumina paired-end technology (2 × 250 bp) generating 756 Mb of data and 709,435 paired reads, and (iii) a Roche GS FLX 8-kb-long paired-end technology generating 103,198 reads with 35,043,425 bp of a raw sequence. *De novo* hybrid assembly was performed using the MaSuRCA genome assembler version 2.1.0 (7), resulting in 5 scaffolds of >1,000 bp. Gaps were initially filled *in silico* using the GapFiller software version 1.3 (8), and the remaining gaps were verified using PCR/sequencing (9).

The *E. albertii* KF1 genome consists of a 4,701,875-bp chromosome with a G+C content of 49.7% and four plasmids (3.8 kb, 5.5 kb, 77 kb, and 85 kb). The annotation was performed using the RAST server (10) and manually inspected for start codons and potential pseudogenes. The chromosome contains 4,422 open reading frames (ORFs), 85 tRNAs, and 7 rRNA operons. In addition, 121 genes are currently designated pseudogenes. The preliminary analysis showed the presence of genes of the locus of enterocyte effacement (LEE), which contains the intimin gene (*eae*) and encodes other virulence factors, the cytolethal distending toxins CdtA/CdtB and invasion protein IbeA. The detailed report of a full analysis of plasmids will be presented in a future publication.

The work will provide a platform to identify new genes that may contribute to this pathogen’s virulence or markers useful in reliable identification of *E. albertii* strains. Moreover, it may help to expand our knowledge about genomic variability and plasticity and the mechanisms of evolution of enteropathogenic *Escherichia* strains.

### Nucleotide sequence accession number.

The annotated chromosome sequence of *E. albertii* KF1 has been deposited in Genbank under accession number CP007025.
